# The Effect of ARTP Mutation on the Degradation Capacity of *Citrobacter* sp. D03

**DOI:** 10.3390/cimb48080765

**Published:** 2026-07-27

**Authors:** Pengji Zhou, Xingyu Liu, Bingqi Li, Lili Yang, Xianya Wu, Xizi Long, Fei Yang

**Affiliations:** Hunan Province Key Laboratory of Typical Environmental Pollution and Health Hazards, School of Public Health, Hengyang Medical School, University of South China, Hengyang 421001, China; zhoupengji@usc.edu.cn (P.Z.); liuxingyu2024@126.com (X.L.); libingqi050418@163.com (B.L.); yanglili3029@163.com (L.Y.); wuxiaoya1229@163.com (X.W.); xizi_long@163.com (X.L.)

**Keywords:** microcystin, *Citrobacter* sp., anaerobic degradation, ARTP mutagenesis

## Abstract

To address the issues of low degradation efficiency in anaerobic bacteria targeting microcystin-LR (MC-LR) and the limitations of traditional genetic modification methods, this study applied ARTP mutagenesis technology for the first time to genetically engineer anaerobic MC-LR-degrading bacteria using the *Citrobacter* sp. D03 isolated from Lake Taihu sediments as the parental strain. Through three rounds of ARTP-induced mutagenesis and directed screening, a highly efficient mutant, C29, was obtained. The results indicate that the optimal mutagenesis conditions are 60 s, with a lethality rate of 90.15%; the anaerobic degradation efficiency of MC-LR by mutant strain C29 was 14% higher than that of the wild-type strain, and the genetic efficiency remained stable after consecutive passages. Whole-genome resequencing of the new strain C29 revealed 10 mutation sites, involving key genes such as *toxD*, *cheA*, *murI*, and *mobB*. qRT-PCR validation showed that the expression of genes related to sulfur metabolism, cell wall synthesis, molybdenum cofactor synthesis, and environmental adaptation was significantly upregulated. The study confirms that ARTP mutagenesis can effectively enhance the degradation capacity of anaerobic degrading bacteria toward MC-LR through polygenic synergistic regulation, providing both microbial resources and a theoretical basis for in situ anaerobic bioremediation of MC-LR pollution in eutrophic water bodies.

## 1. Introduction

In research on the remediation of microcystin-LR (MC-LR) contamination, while it is certainly crucial to thoroughly investigate the molecular mechanisms of gene regulation during bacterial degradation of MC-LR, improving the efficiency of bacterial degradation of MC-LR is equally important and should not be overlooked. Bacteria capable of degrading microcystins often exhibit strain-specific characteristics. In certain aquatic environments, the number of microcystin-degrading bacteria is scarce, and their degradation capacity is inadequate [1]. Conducting in-depth research on the feasibility of transferring strain-specific degradation capabilities, as well as enhancing degradation capacity through genetic modification, has become a critical area requiring urgent breakthroughs in this field.

Current research on the modification of microcystin-degrading genes has primarily focused on the *mlr* gene cluster in aerobic degrading strains, particularly *mlrA* [2,3]. Through the construction of recombinant expression vectors, the *mlrA* gene has been successfully heterologously expressed in hosts such as *Escherichia coli*, *yeast*, and *Saccharomyces cerevisiae* [2,4]. Whole-cell biocatalysts prepared from these recombinant strains demonstrated significantly higher MC-LR removal efficiency than crude enzyme extracts [5]. These findings lay the foundation for the degradation modification of non-*mlr* gene clusters.

Anaerobic degrading bacteria of microcystins exhibit complex genetic regulation, and since the specific degradation mechanisms remain unclear, traditional genetic engineering methods may not be suitable. A combination of random mutagenesis and targeted screening may be more suitable for the genetic enhancement of the whole-cell systems of such bacteria [6]. Common mutagenesis methods include chemical mutagenesis, physical mutagenesis, and novel mutagenesis techniques represented by atmospheric and room-temperature plasma (ARTP) technology [7,8,9]. ARTP is a novel and powerful mutagenesis tool that utilizes radiofrequency atmospheric pressure optical discharge plasma jets. It offers advantages such as high mutation rates, good genetic stability, and broad applicability. Capable of altering gene sequences, it serves as an efficient method for rapidly obtaining desired mutant strains [10]. The combination of ARTP mutagenesis and resequencing is widely used as a screening strategy to identify essential genes in cells and organisms. This method addresses the limitation of being unable to accurately localize single-nucleotide variants (SNVs), including single-nucleotide polymorphisms (SNPs) and small-scale insertions or deletions [11]. Previous studies have utilized this technology for the screening of target strains, including in the field of environmental remediation [12]; therefore, this technology can be employed to enhance the degradation capacity of microcystin-degrading bacteria.

Existing research on the anaerobic biodegradation of MC-LR has largely focused on the isolation and identification of wild-type strains and the characterization of their degradation properties. Zhang et al. studied the anaerobic bacterium *Citrobacter farmeri* A4, isolated from Lake Tai sediment and capable of degrading MC-LR, and found that its anaerobic degradation rate was 0.486 μg/mL/d. This strain did not carry the *mlrABCD* gene cluster and belongs to a non-*mlr* degradation pathway [13]. The anaerobic degrading bacterium *Enterobacter* sp. YF3, discovered by Huang et al., also exhibited low degradation activity (0.34 μg/mL/d) and similarly did not rely on the *mlr* genes [14]. However, these wild-type anaerobic bacteria generally suffered from low degradation efficiency and poor environmental adaptability, and there have been no studies to date applying ARTP technology to enhance the performance of anaerobic MC-LR-degrading bacteria.

This study focuses on establishing a bioremediation system for microcystin-LR (MC-LR) contamination under anaerobic conditions. For the first time, ARTP mutagenesis technology was applied to genetically modify bacteria capable of anaerobic degradation of MC-LR. Through a directed screening strategy, the highly efficient degrading strain *Citrobacter* sp. D03 was isolated from a typical contaminated habitat and used as the parent strain. Mutant strains with enhanced degradation performance were obtained through ARTP mutagenesis and multiple rounds of stress screening. By combining genome resequencing, qRT-PCR, and other techniques to elucidate the molecular mechanisms linking mutation sites to enhanced degradation efficiency, this study aims to enrich anaerobic MC-LR-degrading strains, explore the regulatory mechanisms of ARTP-induced mutagenesis on bacterial functions, and provide theoretical and technical support for in situ anaerobic bioremediation of MC-LR pollution.

## 2. Materials and Methods

### 2.1. Experimental Strain

Test strain: *Citrobacter* sp. D03 isolated in this study.

### 2.2. Main Culture Media

NB medium (Haibo Biotechnology, Qingdao, China): peptone 10.0 g/L, beef extract 3.0 g/L, NaCl 5.0 g/L, pH adjusted to 7.2 ± 0.2. MSM medium (Coolaibo Technology, Beijing, China): MgSO_4_·7H_2_O 1.0 g/L; KH_2_PO_4_ 0.5 g/L; K_2_HPO_4_ 4.0 g/L; NaCl 1.0 g/L; CaCl_2_ 0.02 g/L; FeSO_4_ 0.005 g/L; MnCl_2_·4H_2_O 0.005 g/L; ZnCl_2_ 0.005 g/L; CuCl_2_ 0.0005 g/L. The pH was adjusted to 7.0. Solid media: Standard NB medium and MSM medium; 2% agar (Biosharp, Beijing, China) is added prior to autoclaving.

All of the above media must be incubated in an anaerobic workstation for 48 h prior to use to remove dissolved oxygen from the medium.

### 2.3. Isolation and Purification of Microcystin-Degrading Bacterium D03

Sediment samples were collected from Lake Taihu during cyanobacterial blooms and transported to the laboratory under refrigerated conditions. One gram of sediment was suspended in sterile water and incubated at 30 °C with shaking at 120 rpm for 30 min. After standing for 15 min, 0.1 mL of the supernatant was inoculated into 3 mL of NB medium and enriched at 30 °C for 2 days. Subsequently, 0.1 mL of the culture was transferred to MSM medium supplemented with 3 μg/mL MC-LR (Yuanye, Shanghai, China) and incubated at 30 °C for 24 h. After four successive subcultures, 1 mL of the bacterial suspension was collected and serially diluted. Aliquots of different dilutions were spread onto solid MSM agar plates containing MC-LR using the spread-plate method. A pure strain capable of degrading MC-LR was obtained and designated D03. The strain was preserved in glycerol (Aladdin, Shanghai, China) stock at −80 °C for subsequent experiments. All operations were performed in a DG250 anaerobic incubator (Don Whitley Scientific, Bingley, West Yorkshire, UK).

### 2.4. Gram Staining and Morphological Characteristics of Strain D03

Gram staining of strain D03 was performed according to Bergey’s Manual of Determinative Bacteriology [15]. The reagents used were obtained from Gram Stain Kit (Solarbio, Beijing, China). Following smear preparation, fixation, primary staining with crystal violet, rinsing, mordant treatment with iodine solution, rinsing, decolorization with 95% ethanol until colorless, rinsing, counterstaining with safranin, rinsing, and air-drying, the color and cellular morphology of D03 were observed under an oil immersion objective (Motic, Xiamen, China).

### 2.5. Molecular Biological Characterization of Strain D03

Strain D03 was inoculated into beef extract peptone medium and cultured until the OD_600_ reached 0.6. Then, 4 mL of the bacterial culture was centrifuged at 12,000 rpm for 1 min, the supernatant was discarded, and the bacterial cells were retained. A bacterial genomic DNA extraction kit (TIANGEN, Beijing, China; DP302) was used to extract the DNA.

The 16S rRNA sequence of the strain was amplified using the universal primers 27F (5′-AGAGTTTGATCMTGGCTCAG-3′) and 1492R (5′-TACGGYTACCTTGTTACGACTT-3′). The PCR reaction conditions were as follows: 95 °C for 5 min; 94 °C for 1 min, 58 °C for 1 min, 72 °C for 90 s, for 30 cycles; 72 °C for 10 min. The PCR products were sequenced by Sangon Biotech Company (Shanghai, China); the gene sequence was submitted to NCBI for BLAST analysis (2.15.0).

### 2.6. ARTP-Induced Lethality Curve for Strain D03

Strain D03 was cultured to the logarithmic phase, and the cells were harvested, washed three times with physiological saline, and the OD_600_ value was adjusted to 0.6. An aliquot of the bacterial suspension was uniformly spread onto a sterile metal slide, which was then placed at the designated treatment position of the ARTP mutagenesis system. The instrument parameters were set as follows: helium flow rate of 10.0 SLM, input radio frequency power of 100 W, and gas purity of 99.99% [16]. The mutagenesis durations were set as 0 s for the control group and 15 s, 30 s, 60 s, 90 s, and 120 s for the experimental groups, respectively. After treatment, the slide was immediately transferred into an Eppendorf tube containing 1 mL of physiological saline and vortexed for 3 min. The resulting bacterial suspension was serially diluted, spread onto NB solid medium, and incubated upside down at 30 °C until colonies appeared.

The lethality rate corresponding to each mutagenesis duration was calculated using the formula: Lethality rate (%) = (A − B)/A × 100% (where A represents the number of colonies before mutagenesis and B represents the number of colonies after mutagenesis). The ARTP lethality rate curve was plotted with mutagenesis time as the x-axis and lethality rate as the y-axis, based on which the optimal ARTP treatment duration was selected. A bacterial lethality rate of approximately 90% is most favorable for the generation of positive mutant strains. Therefore, the treatment time yielding a stable lethality rate of around 90% was chosen as the standard duration for subsequent mutagenesis.

### 2.7. Screening of Mutant Strains

The mutagenized strains were inoculated into NB medium and cultured anaerobically until the OD_600_ reached 0.6. The cells were harvested by centrifugation at 10,000 rpm for 10 min. To simulate prolonged bacterial exposure to MC-LR in natural environments, the cells were washed twice with sterile MSM medium, resuspended in MSM medium containing 2 μg/mL MC-LR, and acclimated for 24 h.

After acclimation, the cells were recollected and resuspended in MSM medium supplemented with 2 μg/mL MC-LR to establish the experimental group. The control group consisted of MSM medium containing 2 μg/mL MC-LR without bacterial inoculation. Both acclimation and degradation assays were performed in an anaerobic incubator, with three biological replicates for both the experimental and control groups. During the 144 h degradation period, 50 μL samples were aseptically collected every 24 h. All samples were centrifuged at 12,000 rpm for 15 min at 4 °C to obtain supernatants. The concentration of MC-LR in the supernatants was determined using a microcystin-LR (MC-LR) ELISA kit (Ruifan, Shanghai, China; RF13045). For detailed procedures, refer to the kit instructions [13].

During the mutagenesis and screening process, the surviving mutant strains were assessed for MC-LR degradation capacity. In each round, the strain with the highest MC-LR degradation efficiency under anaerobic conditions was selected as the starting strain for the next round of mutagenesis. The target strain was obtained after three consecutive rounds of mutagenesis and screening.

### 2.8. Genetic Stability Assay

The screened target strain was successively subcultured five times. When the fifth-generation culture reached an OD_600_ of 0.6, an aliquot was subjected to an anaerobic MC-LR degradation assay under the same conditions and procedures as described in Section 2.7.

### 2.9. Genomic Sample Preparation

The mutant strain was inoculated into NB liquid medium for enrichment culture. At the logarithmic growth phase, 50 mL of the culture was dispensed into sterile centrifuge tubes. After centrifugation at 8000 rpm for 10 min at 4 °C, the supernatant was discarded to collect approximately 0.3 g of bacterial cell pellet. The cells were washed twice with ice-cold sterile phosphate-buffered saline (PBS, 1×, pH 7.4 ± 0.2; Biosharp, Beijing, China), transferred aseptically to a pre-chilled 1.5 mL Eppendorf tube, sealed with Parafilm, and immediately flash-frozen in liquid nitrogen.

All samples were transported continuously on dry ice and submitted to BGI (Shenzhen, China) for whole-genome resequencing. 

### 2.10. Genome Resequencing and Assembly

Library construction was carried out using the BGI Optimal DNA Library Prep Kit (BGI, Shenzhen, China) [17]. The detailed procedure included the following steps: genomic DNA was fragmented and subjected to fragment size selection. After end repair and 3′-A-tailing, sequencing adapters were ligated to the DNA fragments, and the library was constructed via PCR amplification. After quality inspection, the qualified library was subjected to single-strand generation and circularization to form single-stranded circular DNA. DNA nanoballs (DNBs) were generated by phi29 DNA polymerase-mediated rolling-circle amplification, and immobilized in the microwells of a sequencing chip. Paired-end sequencing based on combinatorial probe-anchor synthesis (cPAS) was performed on the DNBSEQ-T7 sequencing platform (BGI, Shenzhen, China).

Raw sequencing data were subjected to four-step quality control to obtain clean reads: reads with a cumulative proportion of ≥40% bases showing a quality score ≤ 20 were discarded; reads containing ≥10% N-bases were excluded; adapter-contaminated sequences were removed; and duplicate sequence contamination was eliminated.

### 2.11. SNP Detection

Using the genome of *Citrobacter* sp. D03 as the reference sequence, the clean data of each sample were aligned to the reference genome using the Burrows-Wheeler Aligner (BWA) [18,19]. Variant detection and analysis were performed following the optimal pipeline recommended by the official website of the Genome Analysis Toolkit (GATK, https://gatk.broadinstitute.org/hc/en-us), accessed on 20 November 2024. Duplicate reads were removed using Picard tools [20], and local realignment and base quality score recalibration were conducted using GATK [21,22]. Based on the alignment results, evaluation indices including sequencing depth, coverage, and mapping rate of each sample were statistically analyzed. Software used and version: BWA (0.7.10), GATK (v3.4.0).

### 2.12. qRT-PCR Analysis of Gene Expression at Mutated Loci

MC-LR degradation assays were performed using the mutant strain and *Citrobacter* sp. D03. Samples were collected at 0 h and 48 h after the initiation of the experiment, with *Citrobacter* sp. D03 serving as the control group. qRT-PCR was employed to detect the differential gene expression of each strain at different time points.

#### 2.12.1. RNA Extraction

Bacterial cells at 0 h and 48 h were harvested, ground under liquid nitrogen, and total RNA was extracted using the Trizol method (Vazyme, Nanjing, China) [23]. An equal volume of nuclease-free water (Vazyme, Nanjing, China) was used as the blank control. One microliter of RNA sample was taken to determine the absorbance values at 260 nm and 280 nm (reference wavelength) using an ultramicro ultraviolet spectrophotometer (MIULAB, Hangzhou, China). The detection zone of the instrument was washed twice with nuclease-free water between each measurement to avoid interference. Pure RNA samples exhibited an OD_260_/OD_280_ ratio of 1.8–2.0. The 23S/16S rRNA ratio of the extracted RNA was also determined.

#### 2.12.2. cDNA Synthesis

cDNA was synthesized via reverse transcription using the HiScript^®^ II Q RT SuperMix for qPCR kit (Vazyme, Nanjing, China). The reverse transcription program was set as follows: 50 °C for 15 min and 85 °C for 5 s. After reverse transcription, the samples were diluted and directly used for qRT-PCR or stored at −80 °C for later use.

#### 2.12.3. Validation of Transcriptomic Data by qRT-PCR

The qRT-PCR reaction system was prepared strictly on ice according to the specific components and volumes listed in Table 1. The sequences of the forward and reverse primers used were detailed in Table 2. ChamQ Universal SYBR qPCR Master Mix (Vazyme, Nanjing, China) was used as the fluorescent reagent. The qRT-PCR reaction was performed on an ABI 7500 Real-Time PCR System (Applied Biosystems, Foster City, CA, USA).

After preparation of the reaction mixture, it was mixed thoroughly and subjected to qRT-PCR amplification. Reaction conditions: pre-denaturation (95 °C for 30 s), followed by 40 cycles consisting of denaturation (95 °C for 10 s), annealing (60 °C for 30 s), and extension at 72 °C for 45 s. After the reaction is complete, analyze the melting curve to determine whether the specificity of the PCR reaction meets the requirements. The relative mRNA expression levels of each gene were normalized to 16S rRNA as the internal reference, and each sample was analyzed in quadruplicate. The qRT-PCR quantitative results were analyzed using the 2^−ΔΔCt^ method [24]. The results were analyzed using SPSS statistical analysis software (26.0) [25]. Statistical analysis was performed using one-way ANOVA, and *p* < 0.05 was set as the significance level.

## 3. Results

### 3.1. Morphology and Characteristics of Microcystin-Degrading Strain D03

**Figure 1:**
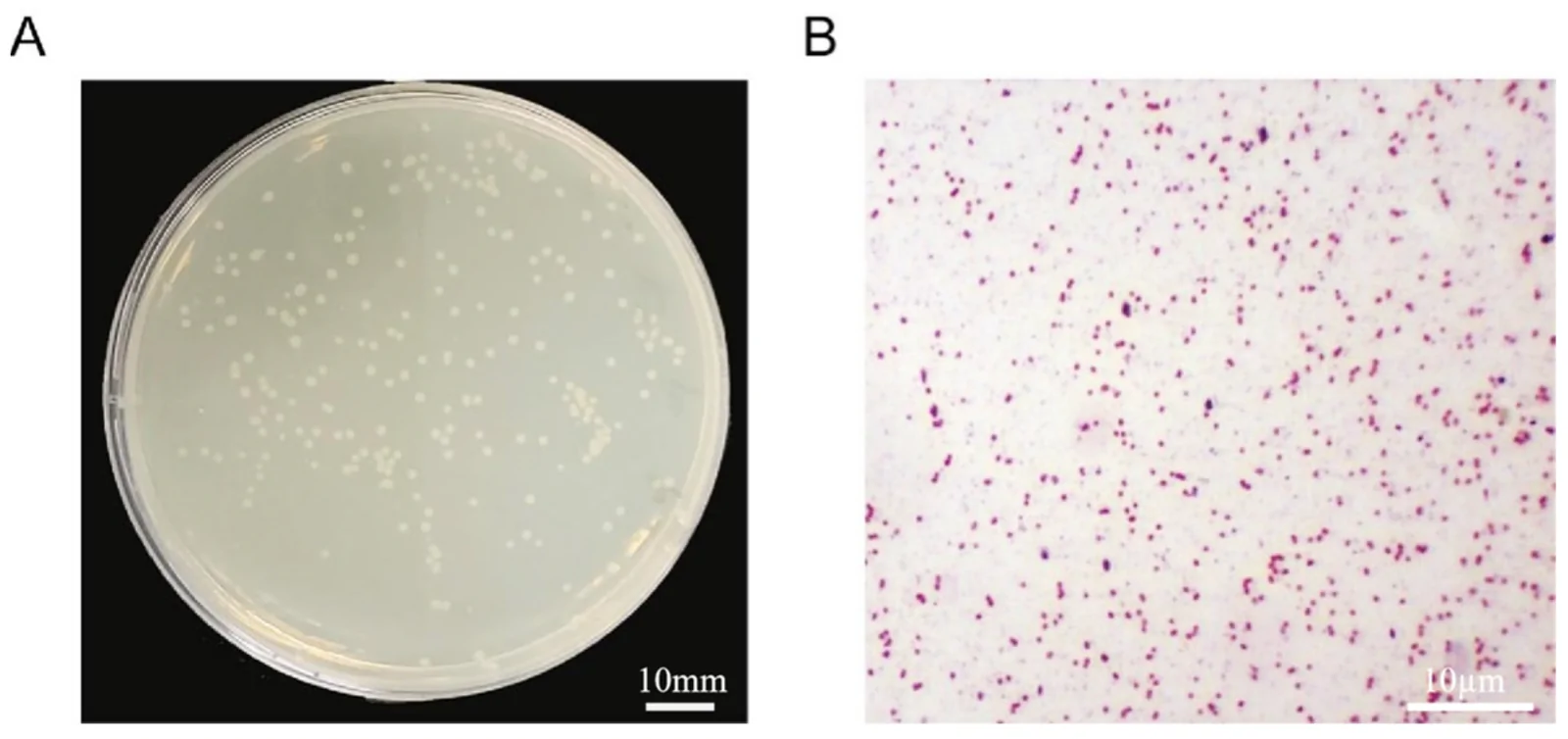
Morphology and characteristics of strain D03: (**A**) solid culture morphological characteristics and (**B**) Gram staining micrograph.

A strain D03 capable of anaerobic MC-LR degradation was isolated from Lake Taihu sediments via repeated selective enrichment culture. This strain exhibited typical colony characteristics on beef extract-peptone agar plates: milky white and opaque, with a smooth and moist surface and regular round margins (Figure 1A). Following standard Gram staining, the cells appeared characteristically pink under light microscopy, confirming that D03 was a Gram-negative bacterium (Figure 1B). Analysis of 16S rDNA sequences showed that strain D03 shares more than 99% sequence similarity with *Citrobacter* sp.

### 3.2. ARTP Lethality Rate Curve of Strain D03

**Figure 2:**
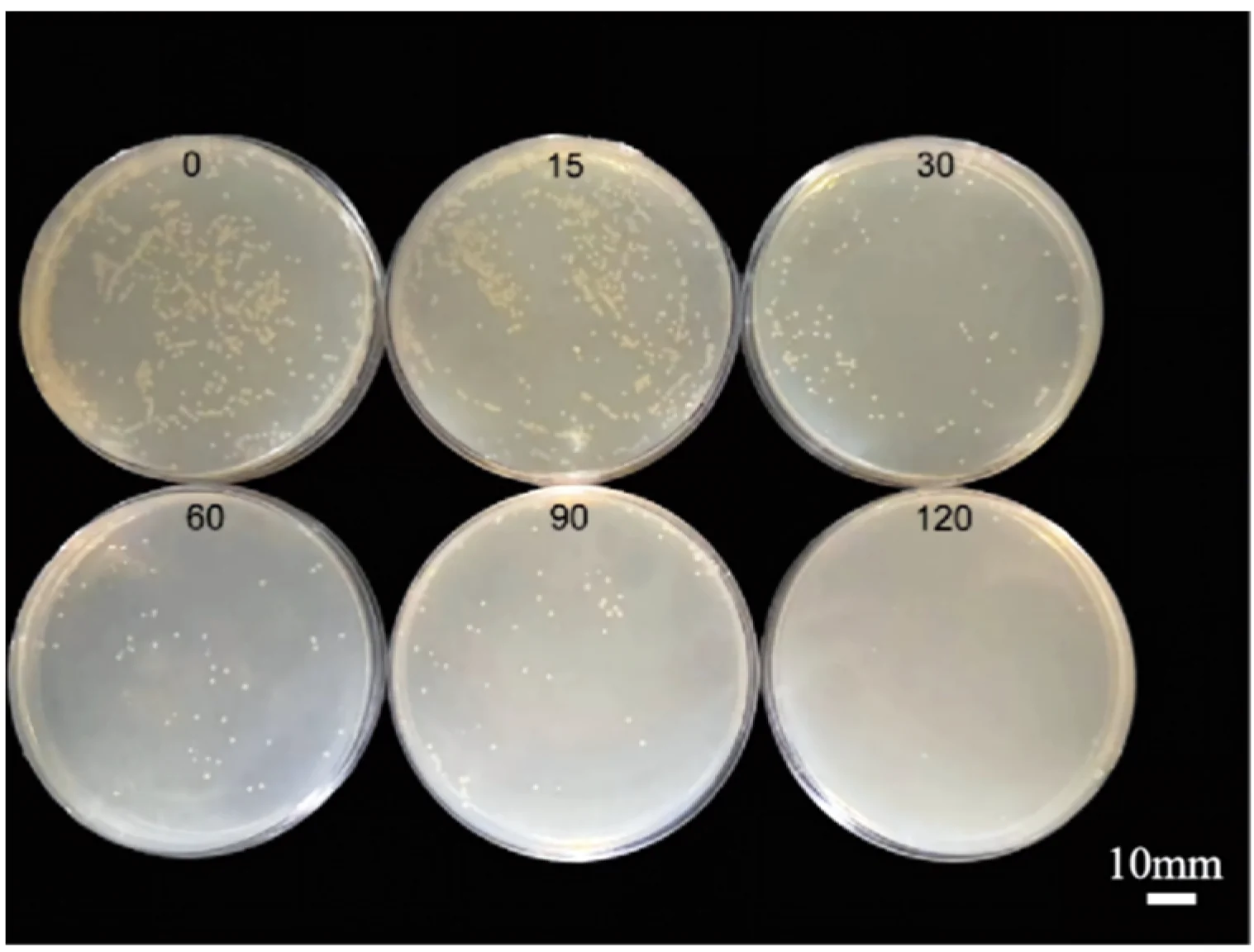
Dilution plating results of bacterial colonies after ARTP mutagenesis at different treatment times (0 s, 15 s, 30 s, 60 s, 90 s, 120 s).

**Figure 3:**
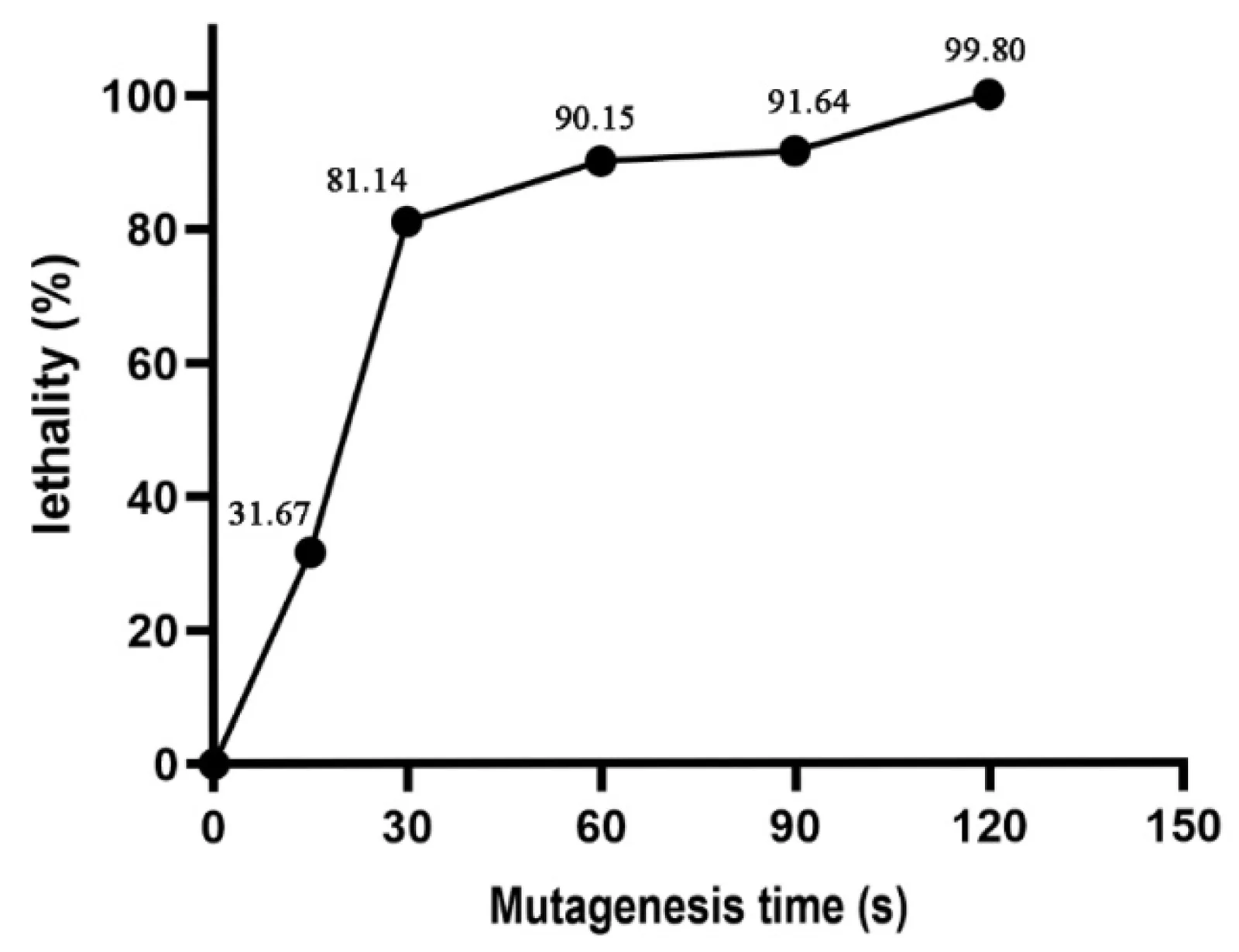
Relationship between ARTP treatment time and lethality rate of D03.

The results of bacterial colony dilution and plating at different ARTP mutagenesis treatment durations (Figure 2) showed that the number of viable bacteria decreased significantly as the treatment duration increased. The results of the first round of lethality curve analysis (Figure 3) further confirmed this trend. The lethality rate was 31.67% after 15 s of mutagenesis and 81.14% at 30 s. The lethality rate of the strain increased to 90.15% at 60 s of mutagenesis, reached 91.64% at 90 s, and was 99.80% at 120 s.

### 3.3. Screening of Mutant Strains

**Figure 4:**
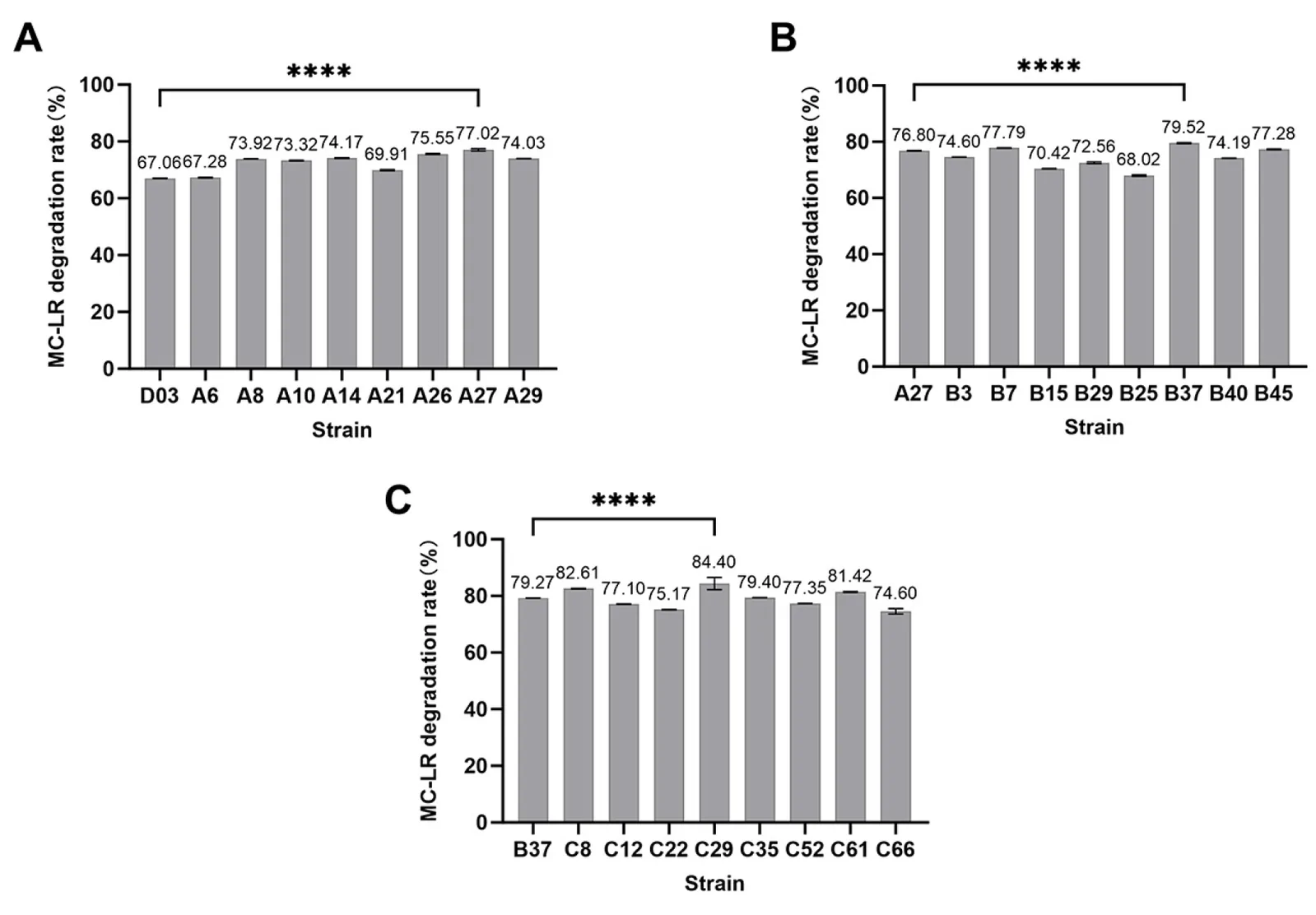
Screening of strains with high MC-LR degradation efficiency by ARTP ((**A**) first round; (**B**) second round; (**C**) third round). The data were presented as the means ± SD of *n* = 3 biologically independent experiments. Statistical analysis was performed using one-way ANOVA by Sidák’s multiple comparisons test. **** *p* < 0.0001.

Based on the results of the relationship between mutagenic lethality and time, a mutagenesis duration of 60 s was selected to perform three rounds of mutagenesis on D03. The mutant strains obtained from each round were subjected to MC-LR degradation assays, and the mutant strain with the best performance was selected for the next round of screening. The starting strains for each round were D03, A27, and B37, respectively. The ELISA standard curve for MC-LR (Figure S1) showed an R^2^ value > 0.999, indicating an excellent fit and high reliability. The degradation results for some mutant strains were shown in Figure 4, where Figure 4A–C represented the MC-LR degradation efficiencies of the three rounds of mutagenized strains, respectively. A total of 21 mutant strains with enhanced degradation capacity were ultimately obtained. Among them, strain C29 exhibited the highest anaerobic degradation capacity for MC-LR.

**Figure 5:**
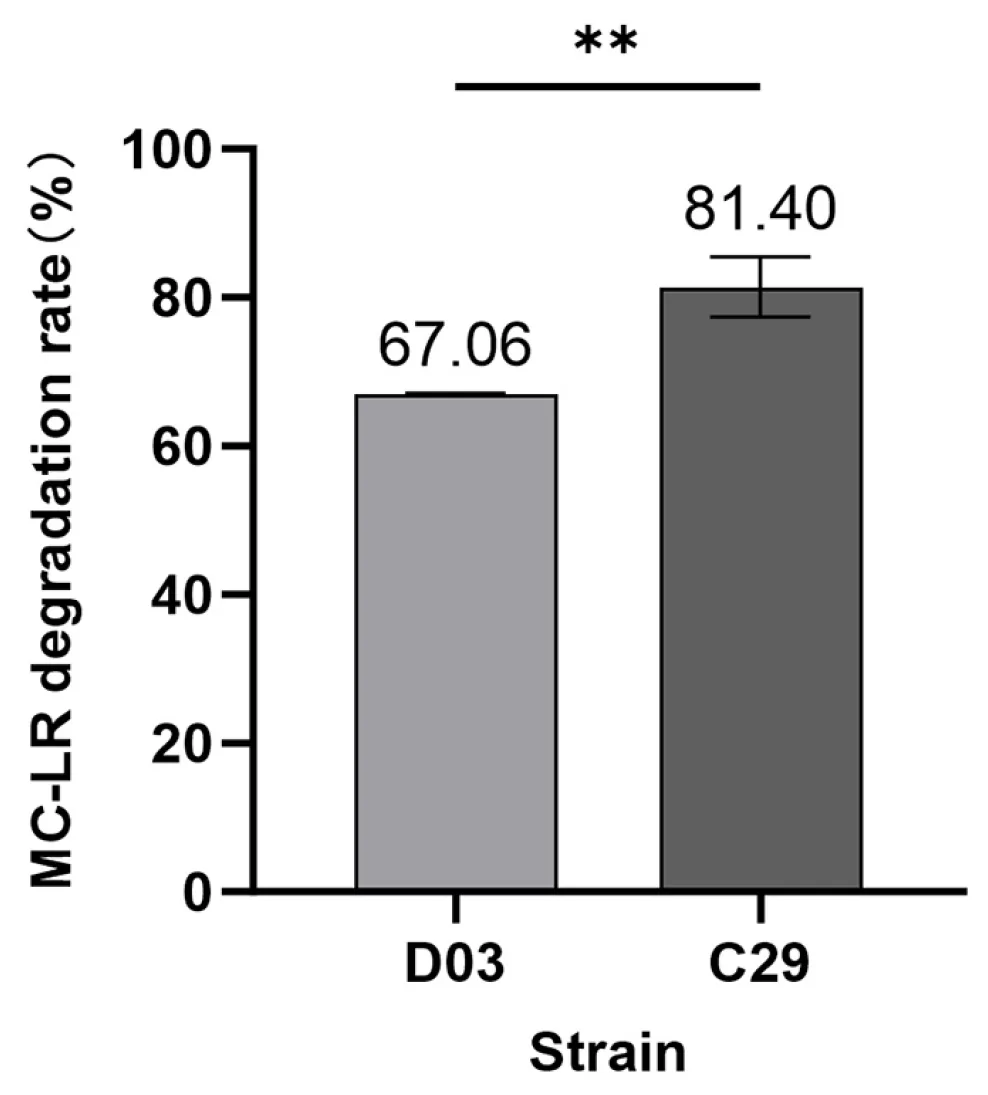
Comparison of the anaerobic degradation efficiency of MC-LR by D03 and C29 at the same time. The data were presented as the means ± SD of *n* = 3 biologically independent experiments. Statistical analysis was performed using unpaired *t*-test. ** *p* < 0.01.

After 5 successive subcultures of strain C29, the degradation assay was repeated, and no significant decrease in degradation efficiency was observed, indicating that the mutant strain exhibited good genetic stability. Over the same time period, its MC-LR degradation efficiency was 14% higher than that of the wild-type strain (Figure 5). The degradation curves for wild-type and mutant strains were shown in Figure S2.

### 3.4. Resequencing of the Mutant Strain

The total reads obtained from sequencing the mutant strain reached 8,728,130. After removing low-quality sequences, adapter sequences and other impurities, 1298 Mb of high-quality clean data were obtained. Analysis of the box-and-whisker plot of sequencing depth distribution (Figure S3) and the line plots of depth at the chromosomal level (Figures S4 and S5) indicated that the overall distribution of sequencing depth for strain C29 was concentrated, with uniform coverage at the chromosomal level. There were no systematic biases or large gaps in coverage, indicating that the sequencing data was of reliable quality. The whole genome of quality-validated strain C29 was compared with that of *Citrobacter* sp. D03, with the alignment results shown in Table 3. The alignment rate was 99.49%, the coverage was 100.00%, and the average sequencing depth reached 99.36×, meeting the requirements for subsequent analysis.

### 3.5. SNP Detection and Annotation Analysis of the Genome

Through alignment analysis, 10 SNP loci were detected in strain C29, among which 3 were non-synonymous mutations within genic regions and 7 were intergenic mutations. The distribution characteristics of SNP loci across genes indicated that all SNPs were single-point mutations in single genes; no gene carried multiple SNP loci, and the mutations were relatively scattered (Figure S6). The specific mutation locations were shown in Table 4.

Based on whole-genome sequencing results, the *toxD* gene encodes a formylglycine-generating enzyme involved in activating sulfatase activity and regulating protein maturation and functional homeostasis. During mutagenesis, a mutation occurred at genomic position 836763, where the nucleotide was changed from G to A, converting the encoded valine to methionine encoded by ATG. The protein encoded by gene *C3GL004277* carried a mutation at genomic position 4466918, in which the nucleotide was altered from T to G, changing the encoded leucine to arginine encoded by CGG. The *cheA* gene harbored a mutation at genomic position 2852311 during mutagenesis, with the nucleotide substituted from G to T, converting the encoded arginine to serine encoded by AGC.

Intergenic mutation sites were analyzed. Mutations at genomic positions 4665367, 4712720, and 4851550 affected the *yjaB*, *murI*, and *mobB* genes, respectively. Mutations at positions 829171, 829186, 3894667, and 4461615 influenced protein products including aldehyde/ketone reductase, transposase, and uncharacterized proteins.

### 3.6. qRT-PCR Validation of Genes at Mutated Loci

**Figure 6:**
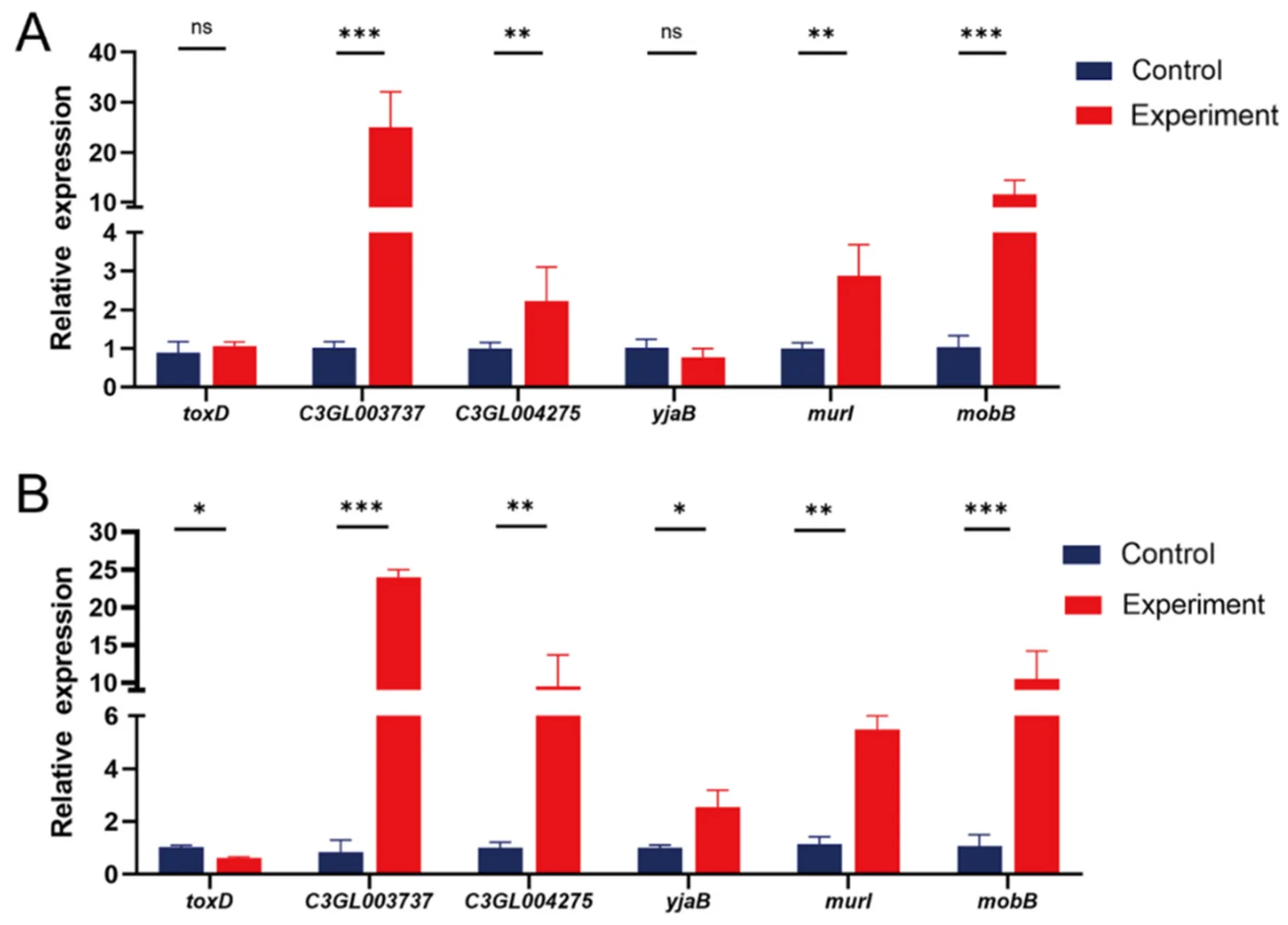
qRT-PCR validation of gene mutation sites (**A**) 0 h; (**B**) 48 h (Mean ± SD; * *p* < 0.05, ** *p* < 0.01, *** *p* < 0.001).

Total RNA was extracted from mutant strain C29 and wild-type strain D03. Combined with the resequencing data, six relevant genes were selected for qRT-PCR analysis. The results showed that compared with the control group, the expression levels of *toxD*, *C3GL003737*, *C3GL004275*, *murI*, and *mobB* were up-regulated by 1.06-, 24.9-, 2.2-, 2.86-, and 11.5-fold at 0 h, respectively, while *yjaB* was down-regulated by 0.61-fold. At 48 h, *C3GL003737*, *C3GL004275*, *yjaB*, *murI*, and *mobB* were up-regulated by 24.1-, 9.48-, 2.53-, 5.5-, and 10.4-fold, respectively, whereas *toxD* was down-regulated by 0.61-fold (Figure 6).

## 4. Discussion

Biodegradation technology holds significant application value in the remediation of MC contamination. Current research aimed at enhancing the efficacy of microcystin-degrading bacteria primarily focuses on optimizing environmental parameters, metabolic regulation strategies, and genetic engineering modifications. Among them, Guo et al. employed response surface methodology to systematically evaluate the mechanisms by which temperature, pH, and initial concentration influence the degradation efficiency of MC-LR by the bacterial community YFMCD4. They established an optimal parameter combination of 30 °C, pH 7, and an initial concentration of 2 μg/mL, under which complete degradation of MC-LR can be achieved within 10 h [26]. Wang et al. found that the degradation efficiency of *Novosphingobium* sp. THN1 toward MC-LR was significantly correlated with the type of carbon source, and that specific substrates could induce changes in the expression of its key degradation genes [27]. The targeted genetic modification based on synthetic biology strategies has become an important approach for optimizing the performance of degradation bacteria, with research focusing on the heterologous expression of the *mlrA* gene. For example, expressing the *mlrA* gene from *Sphingosinicella microcystinivorans* B9 in industrial brewing yeast enables the recombinant strain to achieve 83% toxin removal within 120 h [2]. Following the heterologous expression of MlrA in *Synechocystis* sp. PCC 6803, its whole-cell catalytic activity increased threefold compared to that of the natural host [4]. Liu et al. successfully produced MlrA with a purity of over 90% through overexpression in *Escherichia coli* K12 TB1. This enzyme exhibited high stability, a long half-life, and significant environmental adaptability under high-temperature and alkaline conditions [28].

However, the above strategies have certain limitations: environmental parameters optimized in the laboratory are difficult to match with the dynamic and complex conditions of actual contaminated sites; heterologous expression systems face technical bottlenecks such as insufficient host compatibility and gene silencing, which limit the application of traditional genetic engineering methods. Recent studies have identified multistage degradation intermediates of MC-LR in anaerobically enriched bacterial communities from Lake Taihu sediments, revealing that under anaerobic conditions, two different ring-opening sites of MC-LR in Ala-Leu and Arg-Adda were observed, and three new anaerobic degradation products were first identified, including two hexapeptides (MeAsp-Arg-Adda-Glu-Mdha-Ala and Adda-Glu-Mdha-Ala-Leu-MeAsp) and one end-product pentapeptide (Glu-Mdha-Ala-Leu-MeAsp). Linear, ring-opened heptapeptides, tetrapeptides, short-chain peptides, and the toxin-characteristic fragment Adda were also detected simultaneously in the system. Among these, the pentapeptide intermediates were difficult for anaerobic bacterial communities to further degrade and accumulated over the long term within the system, serving as characteristic markers for identifying anaerobic degradation pathways [29]. However, this study detected intermediates only in mixed microbial communities and lacked validation at the level of single anaerobic pure cultures; the anaerobic MC-LR degradation pathway and key functional genes remained to be systematically elucidated.

Therefore, the selection and optimization of MC-LR-degrading bacteria using modern breeding techniques represent an effective solution to overcome these technical bottlenecks. Compared to traditional methods, ARTP is environmentally friendly, simple to operate, and exhibits strong DNA-damaging capabilities, enabling the more convenient and efficient acquisition of target strains. As a new mutagenesis system, ARTP has been widely applied in microbial breeding [30].

In the field of environmental remediation, Sun et al. successfully screened a mutagenized strain, JC-28-UA12, using ultraviolet-air and room-temperature plasma (UV-ARTP) technology. This strain exhibited a 16.18% increase in lignin degradation rate and demonstrated favorable genetic stability [12]. Wang et al. studied *Bacillus amyloliquefaciens* A3, a strain producing lipopeptide surfactants, and used ARTP mutagenesis to induce genetic variation in the strain. In soil column leaching experiments, they successfully screened out mutant strain 1-24, which demonstrated excellent remediation capabilities. This strain was able to alter the activity of relevant enzymes and remove 45.44% of petroleum hydrocarbons, effectively achieving the remediation of petroleum hydrocarbon-contaminated sites, providing new insights and effective methods for addressing petroleum hydrocarbon pollution [31]. Sun et al. used *Chlorella* as the starting strain to construct an anoxic algal-bacterial symbiotic system for treating low-carbon wastewater, and obtained dominant mutant algal strains through ARTP mutagenesis screening. Following mutagenesis, the removal rates of ammonia nitrogen, total nitrogen, total phosphorus, and COD in the algal-bacterial symbiotic system increased by 8.7%, 7.2%, 10.1%, and 1.3%, respectively. Genes related to metabolism and homeostasis were significantly upregulated, demonstrating significant research and application value in the fields of biological treatment of low-carbon wastewater and environmental remediation [32].

ARTP mutagenesis has also made progress in fields such as metabolite synthesis and polymer degradation. Zhuang Xiong et al. used ARTP mutagenesis to genetically modify strain XZ-A, resulting in a 53.65% increase in the degradation efficiency of the mutated strain XZ-60S toward polyethylene microplastics (PE-MPs) [33]. Cheng et al. successfully obtained a phage-resistant *E. coli* mutant with high L-threonine production using ARTP mutagenesis technology [34]. Zhang et al. isolated *Bacillus licheniformis* XS-4 from fermentation broth and subjected it to ARTP mutagenesis, ultimately obtaining the mutant strain MUT80, which exhibited significantly enhanced protease and amylase activities [35]. These studies provide important references for the non-directed mutagenesis of MC-LR anaerobic degraders.

This study is the first to employ ARTP technology to mutagenize MC-LR-degrading bacteria in order to enhance their degradation capacity. As an efficient mutagenesis method, the treatment duration of ARTP technology has been shown to be a key factor in regulating the mutagenic effect [6]. Typically, the mutagenic effect is optimal when the lethality rate ranges from 90% to 95%, as this yields the highest probability of screening for target mutant strains. Different bacterial species exhibit varying responses to ARTP mutagenesis. When mutagenizing D03, it was found that a mutagenesis duration of 60 s resulted in a mortality rate as high as 90.15%, providing favorable conditions for subsequent screening. After three rounds of mutagenesis, mutant strain C29 demonstrated a 14% increase in MC-LR degradation efficiency compared to the wild-type strain under the same time conditions. The generation of mutant strains via ARTP mutagenesis is attributed to the fact that the particles produced by ARTP can significantly alter the physicochemical properties of the cell wall and cell membrane, causing cellular damage. This forces cells to activate the SOS repair mechanism, which operates at a high level of error tolerance, resulting in the generation of multiple mismatch sites during the repair process [36]. To verify the genetic stability of the mutant strain, researchers cultured and passaged the mutant and wild-type strains under identical conditions, monitoring whether differences between the strains changed after multiple passages, typically ranging from 4 to 10 [36,37,38]. In this study, the mutant strain C29 showed no significant decrease in MC-LR degradation efficiency after five passages compared to the first-generation strain, and its efficiency remained higher than that of the wild-type strain, indicating that this strain exhibits good genetic stability.

When simulating actual environments in the laboratory, researchers typically adjust the temperature, pH, and types and concentrations of pollutants in the culture conditions. For example, in ARTP mutagenesis studies of algae, to investigate the environmental adaptability of mutated algae in actual aquatic environments, Cao et al. inoculated equal amounts of lipid-producing algae before and after mutagenesis into culture media with different pH levels and temperatures, and measured the OD values, dry weight, and lipid yield of the algal cells [36]. Bao et al. isolated *Bacillus subtilis* from chromium waste slag. Through acclimatization using Cr (VI) concentration gradients combined with ARTP mutagenesis, they selected and bred highly tolerant mutant strains, increasing the minimum inhibitory concentration (MIC) of chromium from 80 mg/L to 400 mg/L, demonstrating excellent reduction performance for high concentrations of Cr (VI) [39]. C29 was derived from *Citrobacter* sp. subjected to ARTP mutagenesis, a genus of bacteria widely distributed in natural environments such as soil, water bodies, flora and fauna, and the human gut, and exhibits high adaptability to natural aquatic conditions. Although the adaptability and stability of C29 as an MC-LR-degrading bacterium in actual environments still require systematic evaluation through field monitoring experiments, given its existing degradation capacity, genetic stability under laboratory conditions, and the interactions among various microorganisms in actual environments, this strain holds significant value for the remediation of MC-LR contamination.

Genomic resequencing studies indicated that the primary type of variation in the mutant strains is single-nucleotide polymorphisms (SNPs), rather than large-scale insertions or deletions (Figure S7); the enhanced degradation capacity stems from a complex adaptive mechanism involving the coordinated regulation of multiple genes. Among these, the *toxD* gene encodes formylglycine-generating enzyme (FGE), a non-heme iron enzyme belonging to the SUMF1/EgtB/PvdO family. This enzyme oxidizes specific cysteine/serine residues in the sulfatase precursor to generate formylglycine (FGly), thereby activating sulfatase activity. Following gene mutation, the enzyme’s activity may be affected by alterations in its non-heme iron-binding site or catalytic domain, regulating the bacterium’s ability to acquire carbon and sulfur sources and interfering with secondary metabolic pathways (such as the synthesis of iron carriers or antioxidants). The qRT-PCR results from this study showed that this mutation caused a 0.61-fold downregulation of *toxD* gene expression during the late stage of degradation (48 h), indicating that the activation level of downstream sulfatases was weakened to some extent. Metatranscriptomic studies of anaerobic sediment environments have revealed that, under conditions of substrate scarcity and toxic stress, microbial communities actively upregulated the expression of genes associated with sulfur and carbon metabolism as well as electron transfer to compensate for the lack of conventional nutrient sources, thereby enhancing their ability to adapt to and metabolize complex substrates [40]. In this study, as *toxD* expression decreased, both the cell wall integrity gene (*murI*) and the energy metabolism gene (*mobB*) were significantly upregulated, which was consistent with the response mechanisms described above.

Glutamate racemase (MurI) is a key enzyme in the bacterial cell wall biosynthesis pathway, responsible for catalyzing the stereoisomeric conversion between L-glutamate and D-glutamate, thereby providing the essential D-glutamate residues for the peptidoglycan pentapeptide side chains. MurI functional deficiency directly leads to the inhibition of cell wall precursor synthesis (e.g., UDP-MurNAc-pentapeptide), causing structural defects in the bacterial cell wall, which in turn affects cell morphology, stability, division efficiency, and tolerance to environmental stress [41]. Physical studies of the interface using immobilized lipid bilayers (tBLMs) have revealed that MC-LR can directly embed itself in and disrupt the structure of the lipid bilayer, leading to changes in cell membrane permeability and physical damage [42]. The significant upregulation of the *murI* gene in strain C29 promoted the reinforcement of the peptidoglycan scaffold, enabling it to better maintain cell integrity. This robust cell wall effectively delayed the permeabilization damage caused by MC-LR to the cell membrane, providing the mutant strain with longer survival time and greater metabolic activity, thereby enabling it to degrade MC-LR more effectively.

The biosynthesis of molybdenum cofactors for oxidoreductases is carried out by various enzymes in bacteria. Among these, the MobB protein is a key enzyme in the biosynthesis of molybdopterin-guanine dinucleotide (MGD), which serves as an essential cofactor for molybdenum-dependent enzymes (such as nitrate reductase and formate dehydrogenase). Alterations in the gene encoding the MobB protein may force the strain to remodel the electron transport chain and reprogram energy metabolism [43]. Under anaerobic conditions, the respiratory chain driven by molybdenum-dependent enzymes is a highly efficient biochemical pathway that couples nitrogen metabolism with energy production. Previous studies have confirmed that even in environments lacking preferred carbon sources such as lactate, this type of molybdenum-cofactor-dependent anaerobic respiration can still effectively drive the catabolism of nitrogen-containing amino acids by anaerobic bacteria and provide a stable energy source for their growth [44]. Therefore, the *mobB* gene mutation and its remarkable transcriptional upregulation accelerated molybdenum cofactor (MGD) synthesis, which strengthened anaerobic respiratory chain energy metabolism in strain C29 and further facilitated its anaerobic MC-LR degradation.

CheA is not only the central kinase in the bacterial chemotactic signaling system; recent multi-omics studies have further revealed that its functions extend far beyond the regulation of motility. Through whole-cell proteomics and metabolomics analyses, Ganusova et al. found that the chemotaxis core proteins CheA1/CheA4 in *Azospirillum brasilense* could significantly influence nitrogen metabolic pathways—such as nitrate assimilation and nitrogen fixation—by regulating the global metabolic regulator RpoN, and were directly linked to cellular energy metabolism and transcriptional reprogramming [45]. This suggested that changes in the chemotactic system, together with the nutritional stress caused by the *toxD* mutation, may have led to high expression of the *mobB* and *murI* genes. Such synergistic effects of multiple genes collectively enhanced the anaerobic MC-LR degradation efficiency of mutant strain C29.

Genome resequencing also detected mutations in accessory genes associated with metabolic adaptation. Aldehyde/ketone reductases play a key role in the microbial degradation of exogenous toxic substances and in maintaining intracellular redox balance; transposase activity can induce genomic rearrangements [46]. In biological processes, proteins respond to changes in growth and environmental factors through acetylation, phosphorylation, and ubiquitination reactions [47], and post-translational modifications regulate the functions of various proteins. During the mutagenesis process, mutations occurred in the gene encoding the N-acetyltransferase family enzyme YjaB, which may affect the bacteria’s ability to adapt to their environment. Therefore, these mutations may ultimately result in phenotypic adaptations with enhanced degradation capacity by reshaping substrate metabolic networks, strengthening environmental adaptability, and regulating the allocation of metabolic resources.

## 5. Conclusions

This study marks the first application of ARTP technology to anaerobic microcystin-degrading bacteria. After three rounds of ARTP mutagenesis on *Citrobacter* sp. D03, a new strain, C29, was successfully selected. This strain exhibits good genetic stability and high degradation efficiency, with a 14% increase in MC-LR degradation efficiency. Resequencing results revealed mutations at 10 loci in the genome of the mutagenized strain. qRT-PCR results indicated that the enhanced degradation efficiency of MC-LR was achieved by modulating the expression of functional genes involved in bacterial sulfur metabolism regulation, cell integrity, and environmental adaptability. This study aims to provide a new, highly efficient bacterial strain resource for the removal of MC-LR in natural environments, thereby addressing technical bottlenecks of unstable or low degradation efficiency.

## Figures and Tables

**Table 1:** The qRT-PCR reaction system of gene verification in D03.

Reagent	Volume
2 × ChamQ Universal SYBR qPCR Master Mix	10.0 µL
Forward primer (10 µM)	0.4 µL
Reverse primer (10 µM)	0.4 µL
cDNA	2 µL
ddH_2_O	To 20.0 µL

**Table 2:** The qRT-PCR primers for genes related to mutation sites.

Primers	Sequences (5′ to 3′)
16S rRNA	F: AGAGTTTGATCCTGGCTCAG R: TACGACTTAACCCCAATCGC
*toxD*	F: CGAAGAGCGGCGACAGGAA R: ACGCAGCGAAGGCCAAAA
*C3GL003737*	F: CCACAGCCGCTTCATTAT R: ATTCGGTTTGGGCACTTT
*C3GL004275*	F: CCACAGCCGCTTCATTAT R: TGGTGGCGGTGAGTAAAT
*yjaB*	F: TCGCCTTTATGCTTCTGA R: TCCACTTCTGTCCGTTCC
*murI*	F: TTCTGTCGTCATTGCCATAC R: GGGTGTACTCATTTCCCTCTA
*mobB*	F: ACCCGAACTGGATTTAGC R: TCACTGGCAACTGCGATA

**Table 3:** Sequencing depth and coverage statistics of sample with *Citrobacter* sp. D03 as the reference sequence.

Sample Name	Reference Name	Clean Reads	Clean Bases (bp)	Mapping Rate	Coverage	Average Sequencing Depth
C29	*Citrobacter* sp. D03	3,344,202	501,630,300	99.49	100.00%	99.36

**Table 4:** SNP mutation analysis of strain C29.

Mutation Site	Sequence Change	Gene Name	Position	Mutation Type	Functional Description
836763	G > A	*toxD*	genic	nonsynonymous mutation	formylglycine-generating enzyme (FGE)
4466918	T > G	*C3GL004277*	genic	nonsynonymous mutation	hypothetical protein
2852311	G > T	*cheA*	genic	nonsynonymous mutation	chemotaxis protein CheA
829171	T > G	*C3GL000802*	intergenic	/	hypothetical protein
829186	G > T	*C3GL000802*	intergenic	/	hypothetical protein
3894667	A > G	*C3GL003737*	intergenic	/	aldehyde/ketone reductase
4461615	T > C	*C3GL004275*	intergenic	/	transposase
4665367	T > C	*yjaB*	intergenic	/	lysine acetyltransferase (KAT)
4712720	C > A	*murI*	intergenic	/	glutamate racemase
4851550	T > C	*mobB*	intergenic	/	molybdopterin-guanine dinucleotide biosynthesis protein B

## Data Availability

The original contributions presented in this study are included in the article/Supplementary Materials. Further inquiries can be directed to the corresponding author. The genome resequencing of C29 in the study is openly available in BioProject at PRJNA1474772.

## Supplementary Materials

The following supporting information can be downloaded at: https://www.mdpi.com/article/10.3390/cimb48080765/s1.
