# Supplementary material for: The Effect of ARTP Mutation on the Degradation Capacity of Citrobacter sp. D03
Source: Curr Issues Mol Biol. 2026 Jul 27;48(8):765. doi: 10.3390/cimb48080765 (PMC13511643; doi:10.3390/cimb48080765)
Supplement: Supplementary file 1 [file cimb-48-00765-s001.zip › cimb-4372775-supplementary.pdf]

Supplementary Material

The Effect of ARTP Mutation on the Degradation Capacity of *Citrobacter* sp. D03

Pengji Zhou, Xingyu Liu, Bingqi Li, Lili Yang, Xianya Wu, Xizi Long and Fei Yang *

Hunan Province Key Laboratory of Typical Environmental Pollution and Health Hazards, School of Public Health, Hengyang Medical School, University of South China, Hengyang 421001, China

* Correspondence: phfyang@usc.edu.cn

Supplementary Figure List

Figure S1: ELISA calibration curve for MC-LR.

Figure S2: Degradation efficiency of MC-LR by *Citrobacter* sp. D03 and strain C29; Error bars represented standard deviation (n = 3).

Figure S3: Box-and-whisker plot of genome sequencing depth distribution for strain C29.

Figure S4: Sequencing depth distribution of strain C29 reads aligned to the reference genome of *Citrobacter* sp. D03.

Figure S5: Sequencing depth distribution along the self-assembled genome of strain C29.

Figure S6: Frequency distribution of SNP in genes of the reference strain *Citrobacter* sp. D03 for strain C29.

Figure S7: Statistics of insertion/deletion (InDel) variations in strains C29.

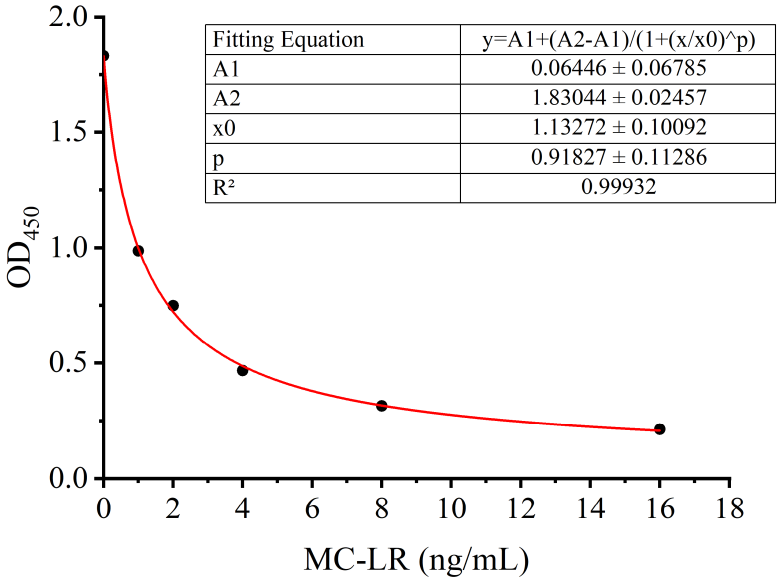

Figure S1: ELISA calibration curve for MC-LR.

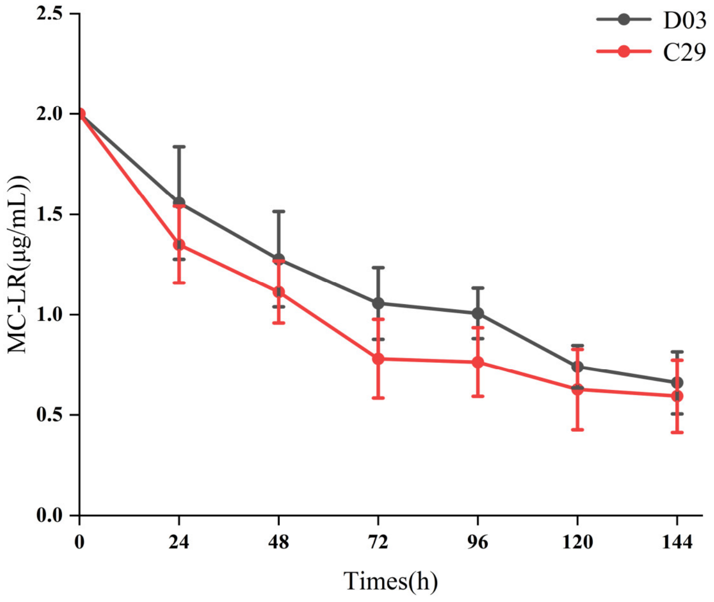

Figure S2: Degradation efficiency of MC-LR by *Citrobacter* sp. D03 and strain C29; Error bars represented standard deviation (n = 3).

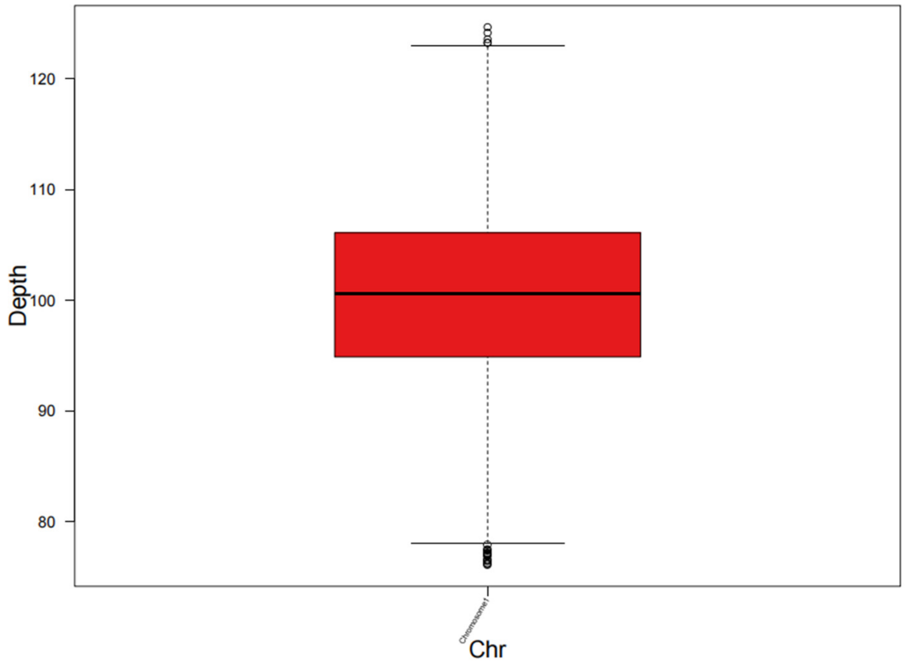

Figure S3: Box-and-whisker plot of genome sequencing depth distribution for strain C29.

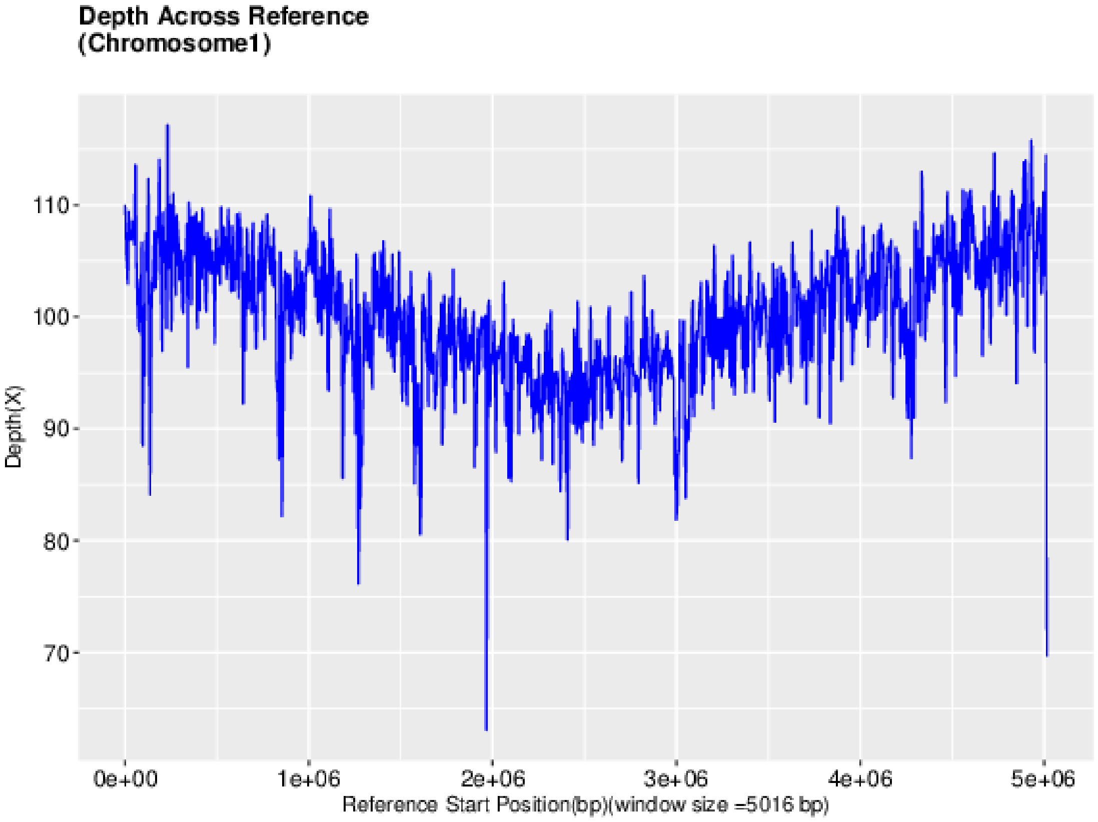

Figure S4: Sequencing depth distribution of strain C29 reads aligned to the reference genome of *Citrobacter* sp. D03.

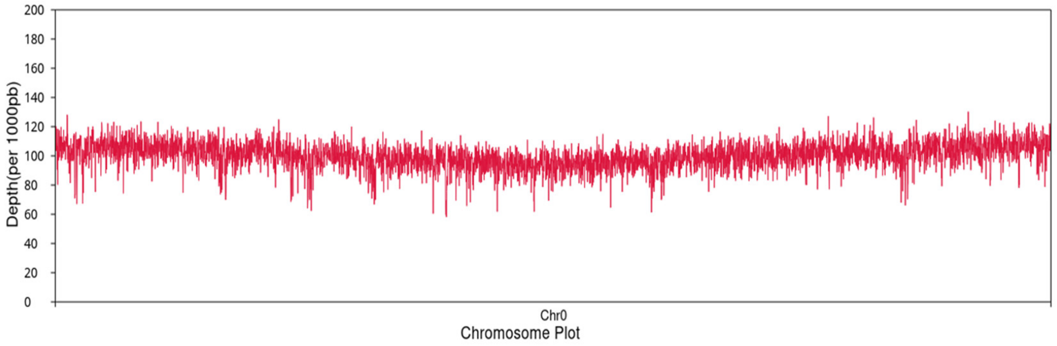

Figure S5 Sequencing depth distribution along the self-assembled genome of strain C29.

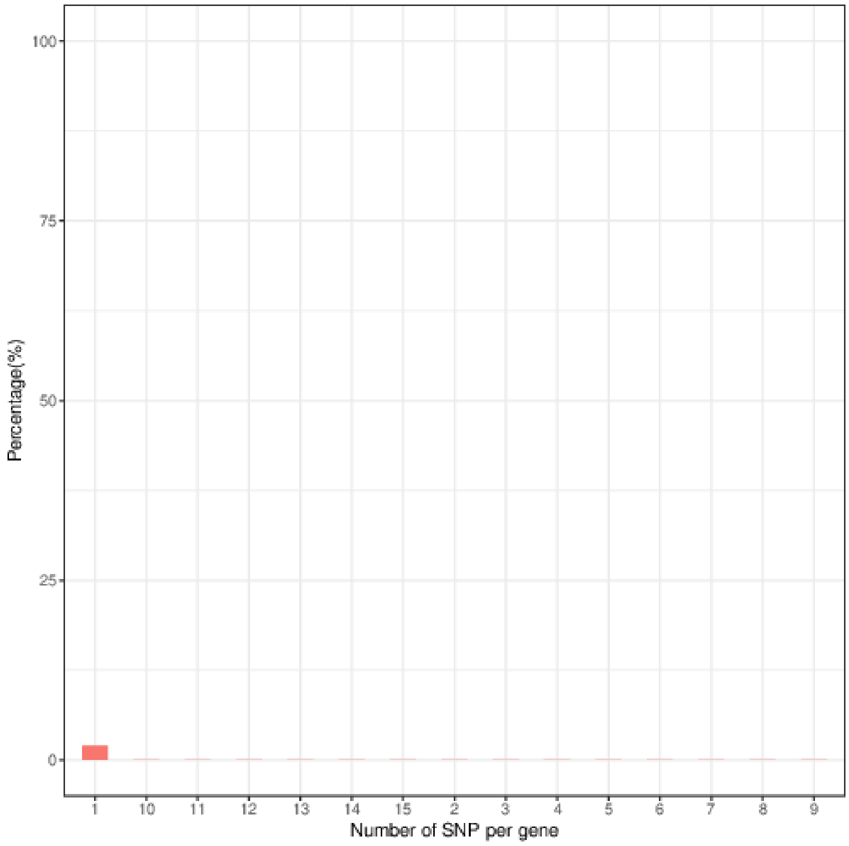

Figure S6: Frequency distribution of SNP in genes of the reference strain *Citrobacter* sp. D03 for strain C29.

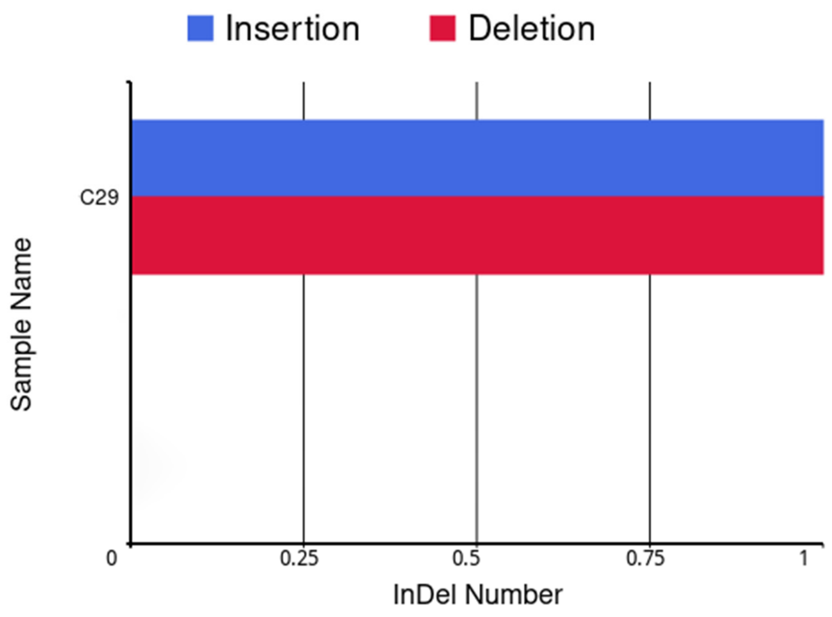

Figure S7: Statistics of insertion/deletion (InDel) variations in strains C29.
